# Intramedullary osteoid osteoma in the humerus of a toddler—A case report and review of the literature

**DOI:** 10.1016/j.radcr.2022.07.073

**Published:** 2022-08-05

**Authors:** Mizuki Hiramatsu, Robert Nakayama, Tomoki Kasahara, Rumi Nakagawa, Toru Hirozane, Sayaka Yamaguchi, Tomoaki Mori, Naofumi Asano, Hajime Okita, Masaya Nakamura, Morio Matsumoto

**Affiliations:** a Department of Orthopaedic Surgery, Keio University School of Medicine, Tokyo, Japan; b Department of Division of Diagnostic Pathology, Keio University School of Medicine, Tokyo, Japan

**Keywords:** Osteoid osteoma, Pediatric, Bone tumor, Osteomyelitis

## Abstract

Osteoid osteoma (OO) is a benign osteoblastic tumor characterized by nocturnal pain that responds well to non-steroidal anti-inflammatory drugs. This condition commonly affects adolescents and young adults, and patients between 5 and 24 years of age account for 85% of all OO cases; it occurs very rarely in patients under 5 years old. Tumors often occur in the cortical bone in the diaphysis and metaphysis of the appendicular skeleton and are more common in the lower extremities than upper extremities. Here, we present an extremely rare case of intramedullary OO that arose in the proximal metaphysis of the humerus in a 2-year-old boy, which mimicked subacute osteomyelitis on imaging studies. We also conducted a retrospective literature review and found that the intramedullary location was fairly common in very young patients (<6 years old) with OO.

## Introduction

Osteoid osteoma (OO) is a benign osteoblastic tumor characterized by nocturnal pain that responds well to non-steroidal anti-inflammatory drugs (NSAIDs). This condition commonly affects adolescents and young adults, and patients between 5 and 24 years of age account for 85% of all OO cases [1]; it occurs very rarely in patients under 5 years old. Tumors often occur in the cortical bone in the diaphysis and metaphysis of the appendicular skeleton and are more common in the lower extremities than upper extremities. Here, we present an extremely rare case of intramedullary OO that arose in the proximal metaphysis of the humerus in a 2-year-old boy, which mimicked subacute osteomyelitis on imaging studies. We also conducted a retrospective literature review and found that the intramedullary location was fairly common in very young patients (<6 years old) with OO.

## Case report

**Fig. 1:**
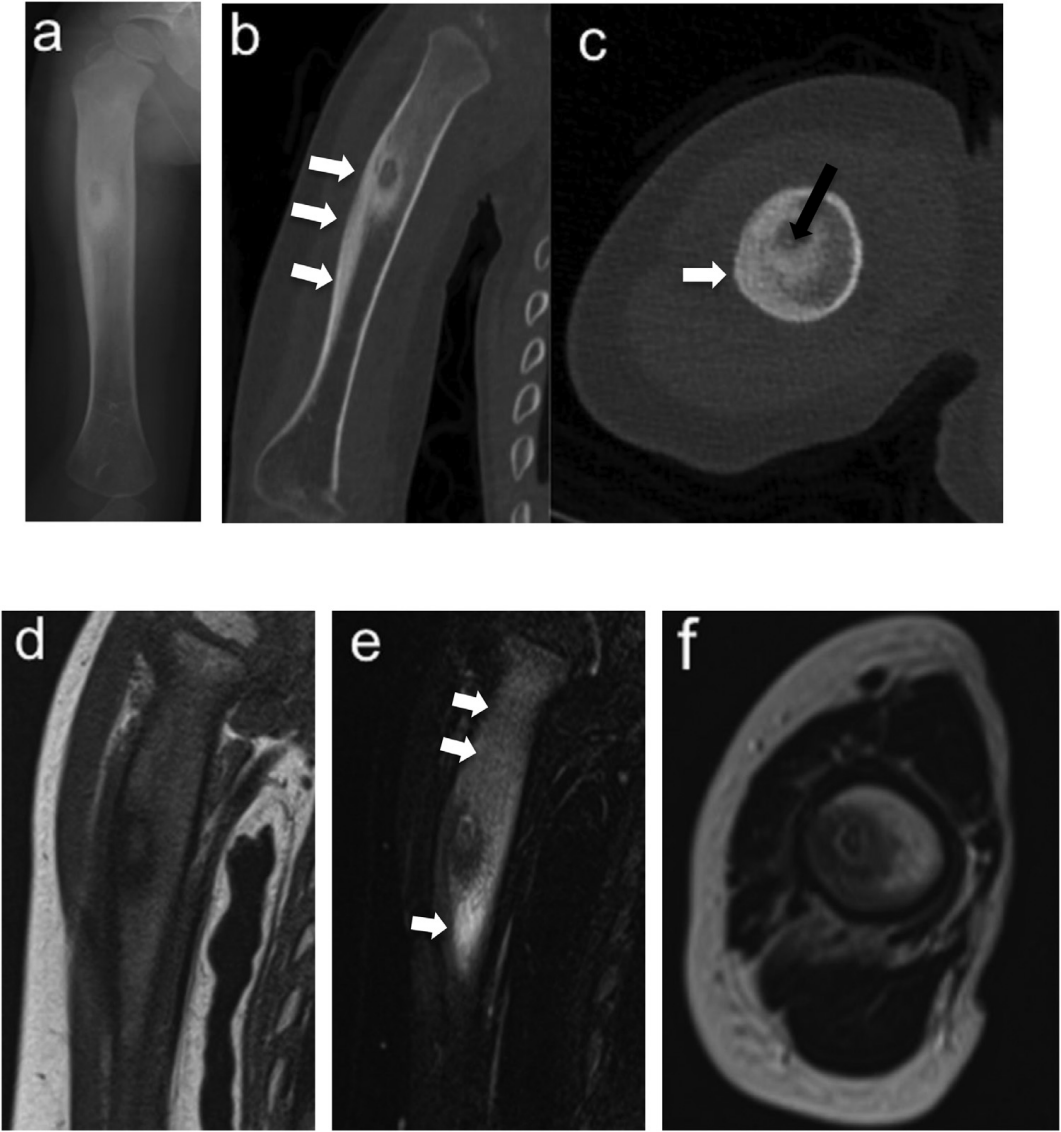
Radiograph, CT, and MRI of the right humerus at the patient's first visit (A: anteroposterior radiograph, B: coronal CT view, C: axial CT view, D: coronal view of T1-weighted MRI, E: coronal view of STIR MRI, F: axial view of T2-weighted MRI). The lucent area was located intramedullary, the nearby bone cortex was unilaterally thickened (white arrows in B, C), and a very fine calcification was observed inside the lucent area (black arrow in C). MRI on STIR showing exceedingly high intensity around the osteosclerotic zone which suggesting bone marrow edema (white arrows in E).

**Fig. 2:**
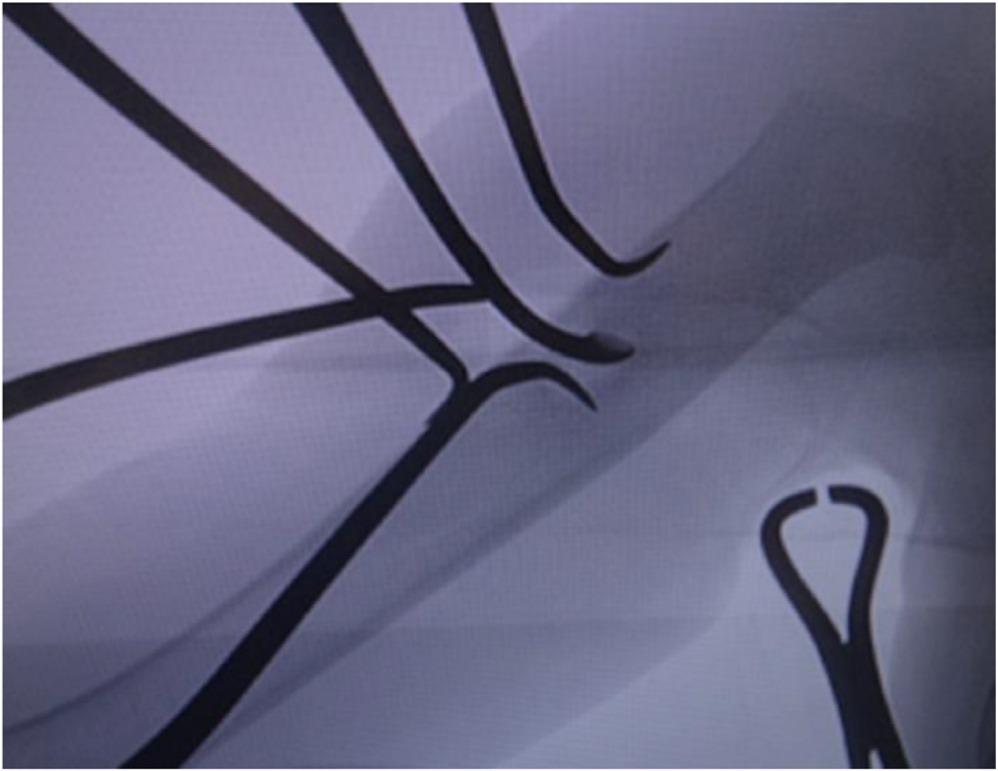
Intraoperative photograph. The operation was completed after confirming that the lucent area could be completely excised by fluoroscopy.

**Fig. 3:**
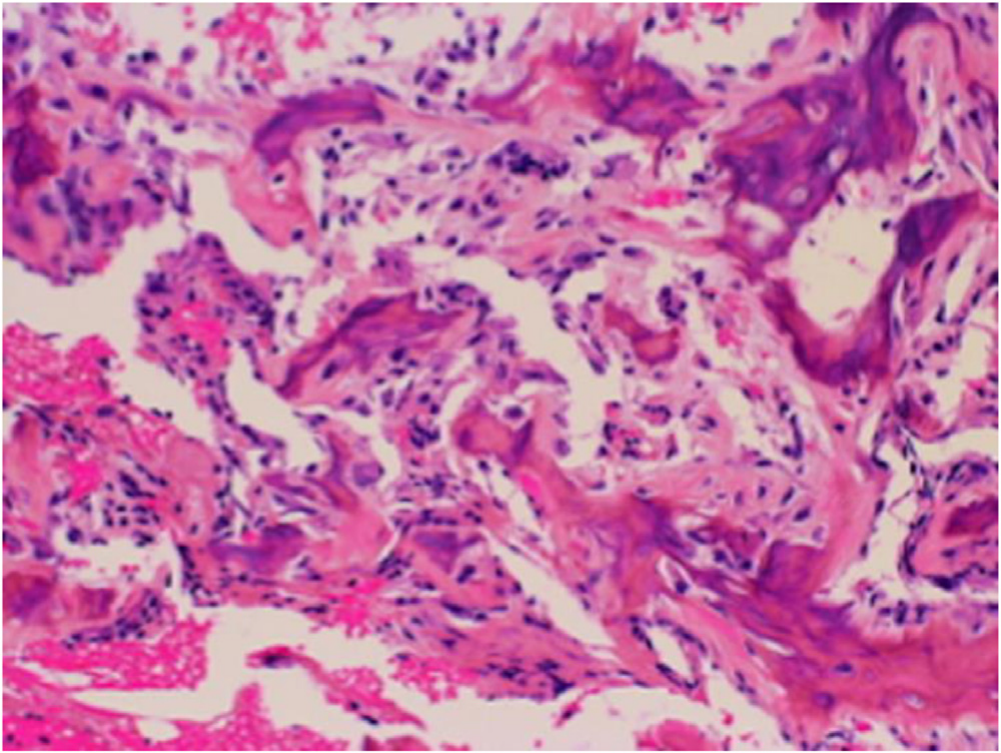
Photomicrograph of the excised tissue. Pathologically, woven bone surrounded by osteoblasts was seen. The cell size was little changed (H&E stain, ×200).

**Fig. 4:**
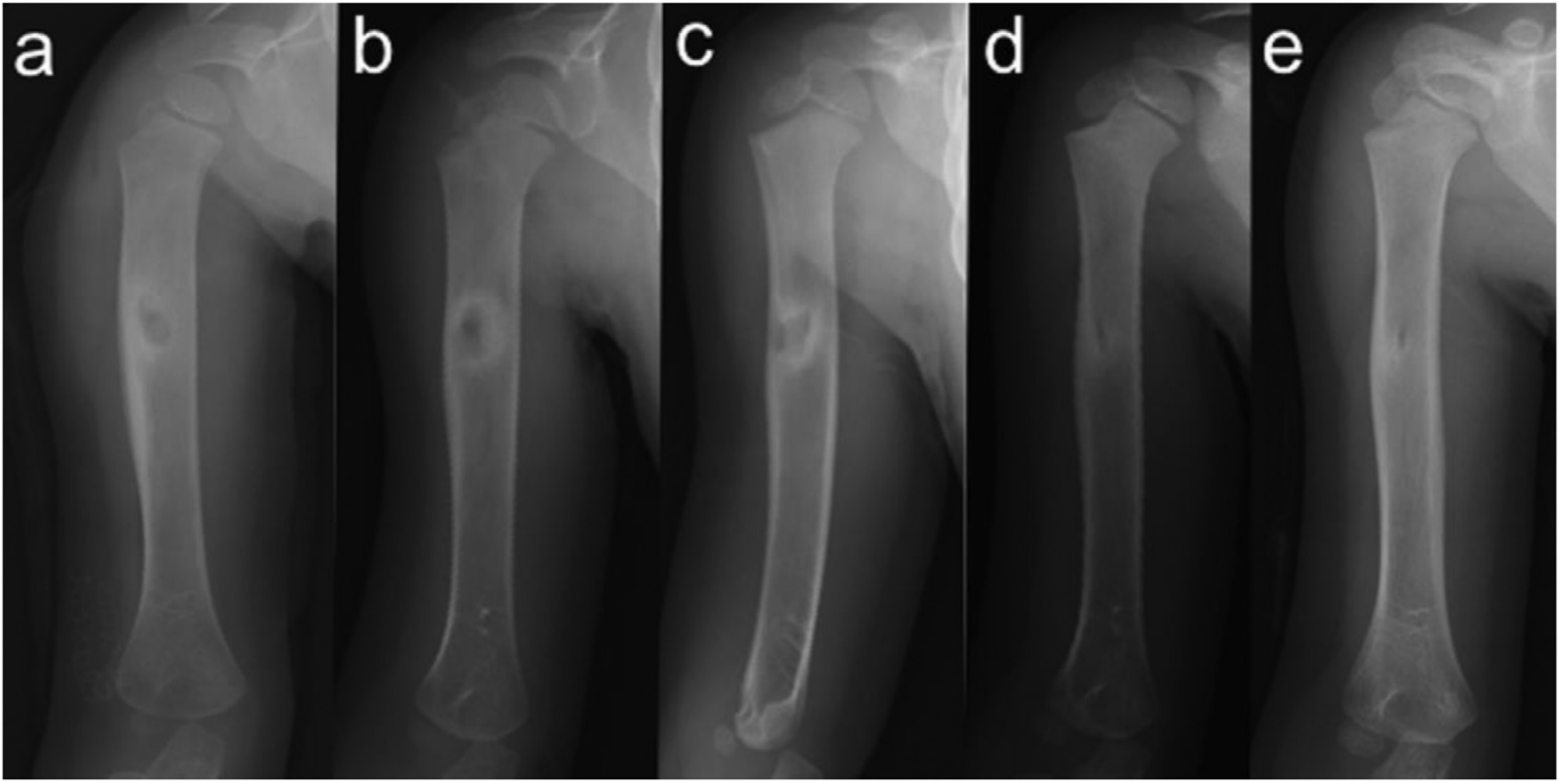
Postoperative anteroposterior radiograph of right humerus taken immediately after surgery (A), 3 months after surgery (B), 6 months after surgery (C), 1 year after surgery (D), and 2 years after surgery (E). The bone lesion resolved over time.

The patient was a boy aged 2 years 9 months with a bone lesion in his right humerus. His parents had noticed reluctance on his part to use his right upper limb for 5 months, and he awoke in discomfort every morning when he visited our hospital. No maternal perinatal, birth, or developmental abnormalities were observed, and there was no notable medical or family history. He had received a Bacille-Calmette-Guérin (BCG) vaccination on his left arm at 7 months. On examination, his upper right limb was thinner than the left, and marked tenderness was observed in the right arm. He used only his left hand when he played with toys did not like to be touched on the right arm. There was no limit to passive range of motion of the shoulder and elbow joints. Plain radiographs of the right humerus showed an 8 × 3 mm oval lucent lesion in the proximal metaphysis surrounded by osteosclerotic bone, and the adjacent cortex was unilaterally thickened (Fig. 1A). On computed tomography (CT), the lucent area was in an intramedullary location, the adjacent cortex was unilaterally thickened, and very fine calcification was observed within the lucent area (Figs. 1B and C). Magnetic resonance imaging (MRI) revealed that the small lucent lesion showed iso-intensity on T1-weighted images and iso-intensity to high intensity on short TI inversion recovery (STIR), which was surrounded by diffuse bone marrow edema with exceedingly high intensity on STIR (Figs. 1D-F). Laboratory investigations including inflammatory response (white blood cell count 7.6 × 10^3^/μL, C-reactive protein 0.01 mg/dL) showed no abnormal findings. Given the age, location of the lesion (metaphysis of the humerus), radiological findings, and recent history of BCG vaccination, subacute osteomyelitis, including BCG osteomyelitis, was primarily suspected, while OO was also considered as a differential diagnosis based on the characteristic pain at the time of waking up and the imaging findings. Curettage of the lesion was performed for both diagnostic and therapeutic purposes. Intraoperative findings showed that the lesion comprised red coarse tissue surrounded by highly firm bone without presence of abscess. Considering the high probability of OO, we further cauterized the surrounding hardened bone with an electric scalpel after simple curettage of the lesion (Fig. 2). Pathologically, the presence of the woven bone surrounded by osteoblasts led to the definitive diagnosis of OO (Fig. 3). No acid-fast microbacteria or fungi were found on Ziehl-Neelsen staining and periodic acid-Schiff staining, respectively. Immediately after the operation the right upper arm pain at the time of waking up disappeared, although it took the child about 3 months to regain full activity of his right upper limb. The bone lesion has resolved over time on plain radiographs (Figs. 4A-E), and no recurrence of symptoms has been apparent for 2 years.

## Discussion

OO is a benign osteoblastic tumor, first described by Jaffe in 1935, which comprises approximately 10% of all benign osseous neoplasms and approximately 5% of all primary bone tumors [2]. Recently, it has been suggested that abnormalities in the transcription factor FOS are implicated in the pathogenesis of OO [3]. There is a male preponderance (2:1 male-to-female ratio) [2]. Adolescents and young adults are more commonly affected, it is very rare in individuals younger than 5 or older than 40 years, and there are reports that 85% of these tumors occur in people aged between 5 and 24 years [1]. According to the bone tumor registry in Japan from 2016 to 2019 compiled by the Japan Orthopedic Association, OO in patients under 5 years of age is very rare, accounting for 2.6% (28 of the total 1,069 OO cases). Tumors often occur in the cortical bone in the diaphysis and metaphysis of the appendicular skeleton, and are more common in the lower extremities than upper extremities. Approximately 50%-60% of lesions occur in the femur and tibia [2]. The characteristic nocturnal pain that responds well to NSAIDs gradually increases over several months to years.

OO characteristically appears as a lucent area called a nidus, with a surrounding reactive sclerotic bone and unilateral thickening of the cortical bone on radiographs and CT [1]. The size of the nidus is usually less than 2.0 cm, and more than half of OOs have calcification within the nidus [4]. MRI is not the best modality for diagnosis because of the difficulty in visualizing the nidus, which shows up as a lesion with iso-intensity on T1-weighted images and a variety of signals on T2-weighted images, while the surrounding sclerotic bone appears as a low signal on both T1- and T2-weighted images. More characteristically to OO, the surrounding bone marrow, soft tissues, and adjacent joints appear with diffuse high signal on fat-suppressed T2-weighted images and STIR [5], which reflect extensive edema. Some reports suggest that younger patients demonstrate a higher degree of bone marrow edema [6].

Kayser et al. classified OO into 4 subtypes according to the location of nidus on axial images of CT or MRI, namely subperiosteal type, cortical type, endosteal type, and intramedullary type [7]. Cortical and subperiosteal types are the most common, accounting for 95% in total, followed by intramedullary type (5%) [7]. Intramedullary types such as we encountered in the present case typically occur in the intra-articular regions, such as femoral neck, spine, and phalangeal bones in the hands and feet [8], and are less common in the metaphysis and diaphysis of the long bones. In addition, the sclerotic change around the nidus has been shown to be milder in the intramedullary type than in the cortical type [9]. By contrast, the intramedullary OO in our patient was found in the metaphysis of the humerus accompanying a marked sclerotic change. These peculiar findings made it difficult for us to rule out other infectious diseases.

Given the above-mentioned characteristic features, image findings of OO are similar to osteomyelitis. Above all, brodie abscesses occur predominantly at the metaphysis of the long bone canals in children, but often have a poorly elevated inflammatory response. Radiography shows lucent lesions with surrounding sclerotic margins in the marrow, very similar to an intramedullary OO. Typical MRI findings of Brodie abscess are indicated by the presence of high-intensity granulation tissue between central rotting/abscess and surrounding osteosclerosis, which shows low intensity on T1-weighted images. This layered appearance is called the penumbra sign. The specificity is 99% and the diagnostic value is high, but the sensitivity is 70%, and it is considered a good possibility that the initial image of Brodie abscess will show the MRI signal pattern, as in our case, without showing the penumbra sign [10].

Given that BCG osteomyelitis is a rare late-onset complication of BCG vaccination that often occurs about 1 year after vaccination, it was one of the predominant differential diagnoses in our case. According to an international survey on the complications of BCG vaccination, the incidence of osteomyelitis was 0.89-2.41 and 0.02-0.06 per 1 million among vaccine recipients at ages <1 year and ≥1 year, respectively [11]. Only a few cases without any immunodeficiency have been reported annually in Japan, even though before age 3 years more than 90% of Japanese children receive multipuncture percutaneous inoculation with BCG Tokyo 172 strain, the least virulent substrain [12]. BCG osteomyelitis is found most commonly in the diaphysis and metaphysis of the long bones. Typically, radiography shows a lucent area and MRI shows a high signal on T2-weighted images, which reflects an abscess [13,14].

Although it is known that pain arising from OO resolves spontaneously over a long period of time, the analgesic effect of NSAIDs is generally not sufficiently long-lasting, and surgery is performed for analgesic purposes. In children, such as in the present case, surgery is often chosen early because NSAIDs are inadvisable owing to the high risk of side effects, and overgrowth of the affected limb may lead to leg-length differences and bone and joint deformities [15,16]. The surgical procedures include surgical resection of the nidus and CT-guided percutaneous ablation. Surgical resection is performed by either intralesional excision or en bloc resection. Although there is no consistent view on the difference in recurrence rate between the 2 resection procedures, the percutaneous method (primary success rate 80%-94%) seems slightly less effective than the surgical method (primary success rate 95%-100%) [17]. In our patient, as it was difficult to make a definitive diagnosis based on clinical and imaging findings alone, surgical resection of the lesion was performed as a diagnostic and therapeutic measure. Electrocauterizing the surrounding sclerotic bone tissue after curettage was performed as an effective adjuvant treatment.

We conducted a retrospective literature review and summarized 15 cases of OO in children younger than 5 years (Table 1) [16,[18], [19], [20], [21], [22], [23], [24], [25], [26], [27], [28]]. The male/female ratio was 7:3, and all the tumors were located in the lower extremities, similar to the clinical features of typical adult cases of OO. Pediatric OO was characterized by symptoms that included limping, tenderness, swelling, atrophy, infrequent use of limb, mood swings, and crying, as well as typical nocturnal pain as observed in adult cases. Diagnosis was reported to be difficult because of these non-specific symptoms, and it often took several months from onset to the definitive diagnosis. In the current case also, the chief complaint was reluctance to use the affected limb, and it took 6 months from the time the parents had first noticed this until the diagnosis. Therefore, when a little child presents to an outpatient clinic with non-specific symptoms such as being reluctant to use his/her extremity but seem to worsen at night, it is necessary to examine the patient for the possibility of OO despite its rarity.

**Table 1:** Past studies reporting cases of OO in children younger than 5 years.

Author	Age/sex	Chief complaint	Time to diagnosis	Location of lesion	Kayser's classification	Treatment
Habermannd (1974)	8 mos/M	Bad mood, night awakening, not to use affected limb	4 mos	Tibia	Difficult to classify	Resection
Black (1979)	4 yrs/F	Pain particularly severe at night	2 mos	Tibia	Difficult to classify	Resection
Bhat (2003)	2 yrs/F	Pain, limp, swelling	2 dys	Femur	Medullary	Resection
Halanski (2005)	2 yrs/M	Pain	4 mos	Tibia	Medullary	Resection
Ekström (2006)	1 yrs/M	Pain at night, not bear weight on affected limb	5 mos	Femur	Medullary	Percutaneous CT-guided radiofrequency coagulation
Martinez (2008)	2 yrs/M	Limp	2 mos	Femur	Medullary	Resection
Virayavanich (2010)	7 mos/F	Not bear weight on affected limb	4 mos	Femur	Medullary	Percutaneous CT-guided radiofrequency coagulation
McKenzie (2019)	3 yrs/M	Pain, limp	1 yrs	Femur	Medullary	Resection
Laliotis (2019)	18 mos-3 yrs	Pain, limp	38 mos	Femur	Medullary	Resection + radiofrequency ablation
				Femur	Intracortical	Resection + radiofrequency ablation
				Tibia	Medullary	CT-guided resection
				Tibia	Not listed	CT-guided resection
Cotta (2019)	1 yrs/M	Limp, swelling, shortening of affected limb	3 mos	Tibia	Endosteal	Resection
Sahin (2019)	1 yrs/M	Pain, crying at night, not bear weight on affected limb	6 mos	Tibia	Intracortical	Percutaneous CT-guided radiofrequency coagulation
Gupta (2020)	11 mos/M	Crying, swelling, decreased use of affected limb	3 mos	Tibia	Medullary	Resection

Because the lesion was in an intramedullary location with surrounding bone marrow edema, it was very difficult to differentiate it from osteomyelitis preoperatively, and it was not possible to diagnose OO only by imaging. We classified the localization of nidus based on Kayser's classification in past case reports of OO occurring in children younger than 5 years (Table 1). Although some cases were difficult to judge because only radiographs were employed without CT or MRI, at least 60% of evaluable cases (9 of 15) were of the intramedullary type; this proportion was surprisingly high, indicating that the intramedullary type was common in children under 5 years old unlike in youth or adults who manifest many cortical types. In 2011, Falappa et al. suggested a higher percentage of intramedullary OO in children younger than 6 years, namely 27% [29], but the results from our detailed review exceeded the previously reported data. Therefore, the possibility of OO, as well as osteomyelitis, should be considered when a young child presents with a symptomatic intramedullary lesion showing a radiolucent area on radiographs and extensive bone marrow edema on MRI.

In conclusion, we report herein a case of intramedullary OO of the humerus in a 2-year-old boy. Because OO in children younger than 5 years is very rare and the symptoms are diverse, which makes it difficult to diagnose, careful imaging is important on considering the possibility of OO, especially when nocturnal limb pain that worsens in the early morning is reported. Given the many cases of intramedullary type OO in young children that have now been reported to date, when an intramedullary lesion is discovered in such patients, OO is one of the conditions that need to be ruled out together with osteomyelitis.

## Patient consent

When the patient's parents were informed that data concerning the case would be submitted for publication, they provided their consent.
